# The wide spectrum of Covid-19 neuropsychiatric complications within a multidisciplinary center

**DOI:** 10.1093/braincomms/fcab135

**Published:** 2021-06-17

**Authors:** Cécile Delorme, Marion Houot, Charlotte Rosso, Stéphanie Carvalho, Thomas Nedelec, Redwan Maatoug, Victor Pitron, Salimata Gassama, Sara Sambin, Stéphanie Bombois, Bastien Herlin, Gaëlle Ouvrard, Gaëlle Bruneteau, Adèle Hesters, Ana Zenovia Gales, Bruno Millet, Foudil Lamari, Stéphane Lehericy, Vincent Navarro, Benjamin Rohaut, Sophie Demeret, Thierry Maisonobe, Marion Yger, Bertrand Degos, Louise-Laure Mariani, Christophe Bouche, Nathalie Dzierzynski, Bruno Oquendo, Flora Ketz, An-Hung Nguyen, Aurélie Kas, Catherine Lubetzki, Jean-Yves Delattre, Jean-Christophe Corvol, CoCo-Neurosciences Study Group, Cecile Delorme, Jean-Christophe Corvol, Jean-Yves Delattre, Stephanie Carvalho, Sandrine Sagnes, Bruno Dubois, Vincent Navarro, Celine Louapre, Tanya Stojkovic, Ahmed Idbaih, Charlotte Rosso, David Grabli, Ana Zenovia Gales, Bruno Millet, Benjamin Rohaut, Eleonore Bayen, Sophie Dupont, Gaelle Bruneteau, Stephane Lehericy, Danielle Seilhean, Alexandra Durr, Foudil Lamari, Vanessa Batista Brochard, Catherine Lubetzki, Pascale Pradat-Diehl, Khe Hoang-Xuan, Bertrand Fontaine, Lionel Naccache, Philippe Fossati, Isabelle Arnulf, Alexandre Carpentier, Yves Edel, Gilberte Robain, Philippe Thoumie, Bertrand Degos, Tarek Sharshar, Sonia Alamowitch, Emmanuelle Apartis-Bourdieu, Charles-Siegried Peretti, Renata Ursu, Nathalie Dzierzynski, Kiyoka Kinugawa Bourron, Joel Belmin, Bruno Oquendo, Eric Pautas, Marc Verny, Yves Samson, Sara Leder, Anne Leger, Sandrine Deltour, Flore Baronnet, Stephanie Bombois, Mehdi Touat, Marc Sanson, Caroline Dehais, Caroline Houillier, Florence Laigle-Donadey, Dimitri Psimaras, Agusti Alenton, Nadia Younan, Nicolas Villain, Maria del Mar Amador, Louise-Laure Mariani, Nicolas Mezouar, Graziella Mangone, Aurelie Meneret, Andreas Hartmann, Clement Tarrano, David Bendetowicz, Pierre-François Pradat, Michel Baulac, Sara Sambin, Phintip Pichit, Florence Chochon, Adele Hesters, Bastien Herlin, An Hung Nguyen, Valerie Porcher, Alexandre Demoule, Elise Morawiec, Julien Mayaux, Morgan Faure, Claire Ewenczyk, Giulia Coarelli, Anna Heinzmann, Marion Masingue, Guillaume Bassez, Isabelle An, Yulia Worbe, Virginie Lambrecq, Rabab Debs, Esteban Munoz Musat, Timothee Lenglet, Virginie Lambrecq, Aurelie Hanin, Lydia Chougar, Nathalia Shor, Nadya Pyatigorskaya, Damien Galanaud, Delphine Leclercq, Sophie Demeret, Albert Cao, Clemence Marois, Nicolas Weiss, Salimata Gassama, Loic Le Guennec, Vincent Degos, Alice Jacquens, Thomas Similowski, Capucine Morelot-Panzini, Jean-Yves Rotge, Bertrand Saudreau, Victor Pitron, Nassim Sarni, Nathalie Girault, Redwan Maatoug, Smaranda Leu, Lionel Thivard, Karima Mokhtari, Isabelle Plu, Bruno Gonçalves, Laure Bottin, Marion Yger, Gaelle Ouvrard, Rebecca Haddad, Flora Ketz, Carmelo Lafuente, Christel Oasi, Bruno Megabarne, Dominique Herve, Haysam Salman, Armelle Rametti-Lacroux, Alize Chalançon, Anais Herve, Hugo Royer, Florence Beauzor, Valentine Maheo, Christelle Laganot, Camille Minelli, Aurelie Fekete, Abel Grine, Marie Biet, Rania Hilab, Aurore Besnard, Meriem Bouguerra, Gwen Goudard, Saida Houairi, Saba Al-Youssef, Christine Pires, Anissa Oukhedouma, Katarzyna Siuda-Krzywicka, Tal Seidel Malkinson, Hanane Agguini, Safia Said, Marion Houot

**Affiliations:** 1 Sorbonne Université, Paris Brain Institute – ICM, Inserm U1127, CNRS UMR 7225, Paris, France; 2 Assistance Publique Hôpitaux de Paris, Sorbonne Université, Pitié-Salpêtrière Hospital, Département de Neurologie, Paris, France; 3 Assistance Publique Hôpitaux de Paris, Sorbonne Université, Pitié-Salpêtrière Hospital, Institut de la Mémoire et de la maladie d’Alzheimer, Département de Neurologie, Paris, France; 4 Assistance Publique Hôpitaux de Paris, Sorbonne Université, Pitié-Salpêtrière Hospital, Centre of Excellence of Neurodegenerative Disease (CoEN), Paris, France; 5 Assistance Publique Hôpitaux de Paris, Sorbonne Université, Pitié-Salpêtrière Hospital, Urgences cérébro-Vasculaires, Paris, France; 6 Paris Brain Institute - ICM Stroke network, STAR team, Pitié-Salpêtrière Hospital, Paris, France; 7 INRIA, Aramis project-team, Paris, France; 8 Assistance Publique Hôpitaux de Paris, Sorbonne Université, Pitié-Salpêtrière Hospital, Service de Psychiatrie adulte, Paris, France; 9 Institut Jean-Nicod,UMR 8129, ENS/EHESS/CNRS, IEC, PSL Research University, Paris, France; 10 Assistance Publique Hôpitaux de Paris, Sorbonne Université, Pitié-Salpêtrière Hospital, Unité d’épileptologie, Département de Neurologie, Paris, France; 11 Assistance Publique Hôpitaux de Paris, Sorbonne Université, Pitié Salpêtrière Hospital, Département de Médecine Physique et de réadaptation, Paris, France; 12 Assistance Publique Hôpitaux de Paris, Sorbonne Université, Rothschild Hospital, Service de Neuro-orthopédie, Paris, France; 13 Sorbonne Université, Institut National de la Santé et de la Recherche Médicale, Association Institut de Myologie, Centre de Recherche en Myologie, UMRS974, Paris, France; 14 Assistance Publique Hôpitaux de Paris, Sorbonne Université, Pitié-Salpêtrière Hospital, Service des Pathologies du sommeil, Paris, France; 15 Assistance Publique Hôpitaux de Paris, Sorbonne Université, Pitié-Salpêtrière Hospital, Département de Biochimie Métabolique; Paris, France; 16 Assistance Publique Hôpitaux de Paris, Sorbonne Université, Pitié-Salpêtrière Hospital, Département de Neuroradiologie, Paris, France; 17 Assistance Publique Hôpitaux de Paris, Sorbonne Université, Pitié-Salpêtrière Hospital, Département de Neurophysiologie Clinique, Paris, France; 18 Assistance Publique Hôpitaux de Paris, Sorbonne Université Saint-Antoine Hospital, Unité de Soins intentifs neurovasculaires, Paris, France; 19 Assistance Publique Hôpitaux de Paris, Sorbonne Université, Avicenne Hospital, Département de Neurologie; Bobigny, France; 20 Assistance Publique Hôpitaux de Paris, Sorbonne Université, Tenon Hospital, Département de Psychiatrie, Paris, France; 21 Assistance Publique Hôpitaux de Paris, Sorbonne Université, Pitié-Salpêtrière Charles Foix Hospital, Service de Gériatrie à orientation neurologique, Paris, France; 22 Assistance Publique Hôpitaux de Paris, Sorbonne Université, Charles Foix Hospital, Service de Gériatrie polyvalente, Paris, France; 23 Assistance Publique Hôpitaux de Paris, Sorbonne Université, Pitié-Salpêtrière, Service d’Addictologie, Paris, France; 24 Assistance Publique Hôpitaux de Paris, Sorbonne Université, Pitié-Salpêtrière Hospital, Service de Médecine Nucléaire et LIB, INSERM U1146, Paris, France

**Keywords:** Covid-19, Encephalopathy, Encephalitis, Critical illness neuropathy

## Abstract

A variety of neuropsychiatric complications has been described in association with Covid-19 infection. Large scale studies presenting a wider picture of these complications and their relative frequency are lacking. The objective of our study was to describe the spectrum of neurological and psychiatric complications in patients with Covid-19 seen in a multidisciplinary hospital center over six months.

We conducted a retrospective, observational study on all patients showing neurological or psychiatric symptoms in the context of Covid-19 seen in the medical and university neuroscience department of Assistance Publique Hopitaux de Paris -Sorbonne University. We collected demographic data, comorbidities, symptoms and severity of Covid-19 infection, neurological and psychiatric symptoms, neurological and psychiatric examination data and, when available, results from CSF analysis, MRI, EEG and EMG.

A total of 249 Covid-19 patients with a *de novo* neurological or psychiatric manifestation were included in the database and 245 were included in the final analyses. One-hundred fourteen patients (47%) were admitted to the intensive care unit and 10 (4%) died. The most frequent neuropsychiatric complications diagnosed were encephalopathy (43%), critical illness polyneuropathy and myopathy (26%), isolated psychiatric disturbance (18%), and cerebrovascular disorders (16%). No patients showed CSF evidence of SARS-CoV-2. Encephalopathy was associated with older age and higher risk of death. Critical illness neuromyopathy was associated with an extended stay in the intensive care unit.

The majority of these neuropsychiatric complications could be imputed to critical illness, intensive care and systemic inflammation, which contrasts with the paucity of more direct SARS-CoV-2-related complications or post-infection disorders.

## Supplementary Material

fcab135_Supplementary_Data

