# The wide spectrum of COVID-19 neuropsychiatric complications within a multidisciplinary centre

**DOI:** 10.1093/braincomms/fcab135

**Published:** 2021-06-17

**Authors:** Cécile Delorme, Marion Houot, Charlotte Rosso, Stéphanie Carvalho, Thomas Nedelec, Redwan Maatoug, Victor Pitron, Salimata Gassama, Sara Sambin, Stéphanie Bombois, Bastien Herlin, Gaëlle Ouvrard, Gaëlle Bruneteau, Adèle Hesters, Ana Zenovia Gales, Bruno Millet, Foudil Lamari, Stéphane Lehericy, Vincent Navarro, Benjamin Rohaut, Sophie Demeret, Thierry Maisonobe, Marion Yger, Bertrand Degos, Louise-Laure Mariani, Christophe Bouche, Nathalie Dzierzynski, Bruno Oquendo, Flora Ketz, An-Hung Nguyen, Aurélie Kas, Catherine Lubetzki, Jean-Yves Delattre, Jean-Christophe Corvol, CoCo-Neurosciences Study Group, Cecile Delorme, Jean-Christophe Corvol, Jean-Yves Delattre, Stephanie Carvalho, Sandrine Sagnes, Bruno Dubois, Vincent Navarro, Celine Louapre, Tanya Stojkovic, Ahmed Idbaih, Charlotte Rosso, David Grabli, Ana Zenovia Gales, Bruno Millet, Benjamin Rohaut, Eleonore Bayen, Sophie Dupont, Gaelle Bruneteau, Stephane Lehericy, Danielle Seilhean, Alexandra Durr, Foudil Lamari, Vanessa Batista Brochard, Catherine Lubetzki, Pascale Pradat-Diehl, Khe Hoang-Xuan, Bertrand Fontaine, Lionel Naccache, Philippe Fossati, Isabelle Arnulf, Alexandre Carpentier, Yves Edel, Gilberte Robain, Philippe Thoumie, Bertrand Degos, Tarek Sharshar, Sonia Alamowitch, Emmanuelle Apartis-Bourdieu, Charles-Siegried Peretti, Renata Ursu, Nathalie Dzierzynski, Kiyoka Kinugawa Bourron, Joel Belmin, Bruno Oquendo, Eric Pautas, Marc Verny, Yves Samson, Sara Leder, Anne Leger, Sandrine Deltour, Flore Baronnet, Stephanie Bombois, Mehdi Touat, Marc Sanson, Caroline Dehais, Caroline Houillier, Florence Laigle-Donadey, Dimitri Psimaras, Agusti Alenton, Nadia Younan, Nicolas Villain, Maria del Mar Amador, Louise-Laure Mariani, Nicolas Mezouar, Graziella Mangone, Aurelie Meneret, Andreas Hartmann, Clement Tarrano, David Bendetowicz, Pierre-François Pradat, Michel Baulac, Sara Sambin, Phintip Pichit, Florence Chochon, Adele Hesters, Bastien Herlin, An Hung Nguyen, Valerie Porcher, Alexandre Demoule, Elise Morawiec, Julien Mayaux, Morgan Faure, Claire Ewenczyk, Giulia Coarelli, Anna Heinzmann, Marion Masingue, Guillaume Bassez, Isabelle An, Yulia Worbe, Virginie Lambrecq, Rabab Debs, Esteban Munoz Musat, Timothee Lenglet, Virginie Lambrecq, Aurelie Hanin, Lydia Chougar, Nathalia Shor, Nadya Pyatigorskaya, Damien Galanaud, Delphine Leclercq, Sophie Demeret, Albert Cao, Clemence Marois, Nicolas Weiss, Salimata Gassama, Loic Le Guennec, Vincent Degos, Alice Jacquens, Thomas Similowski, Capucine Morelot-Panzini, Jean-Yves Rotge, Bertrand Saudreau, Victor Pitron, Nassim Sarni, Nathalie Girault, Redwan Maatoug, Smaranda Leu, Lionel Thivard, Karima Mokhtari, Isabelle Plu, Bruno Gonçalves, Laure Bottin, Marion Yger, Gaelle Ouvrard, Rebecca Haddad, Flora Ketz, Carmelo Lafuente, Christel Oasi, Bruno Megabarne, Dominique Herve, Haysam Salman, Armelle Rametti-Lacroux, Alize Chalançon, Anais Herve, Hugo Royer, Florence Beauzor, Valentine Maheo, Christelle Laganot, Camille Minelli, Aurelie Fekete, Abel Grine, Marie Biet, Rania Hilab, Aurore Besnard, Meriem Bouguerra, Gwen Goudard, Saida Houairi, Saba Al-Youssef, Christine Pires, Anissa Oukhedouma, Katarzyna Siuda-Krzywicka, Tal Seidel Malkinson, Hanane Agguini, Safia Said, Marion Houot

**Affiliations:** CNRS UMR 7225, Sorbonne Université, Paris Brain Institute—ICM, Inserm U1127, Paris 75013, France; Département de Neurologie, Assistance Publique Hôpitaux de Paris, Sorbonne Université, Pitié-Salpêtrière Hospital, Paris 75013, France; CNRS UMR 7225, Sorbonne Université, Paris Brain Institute—ICM, Inserm U1127, Paris 75013, France; Département de Neurologie, Assistance Publique Hôpitaux de Paris, Sorbonne Université, Pitié-Salpêtrière Hospital, Institut de la Mémoire et de la maladie d’Alzheimer, Paris 75013, France; Centre of Excellence of Neurodegenerative Disease (CoEN), Assistance Publique Hôpitaux de Paris, Sorbonne Université, Pitié-Salpêtrière Hospital, Paris 75013, France; CNRS UMR 7225, Sorbonne Université, Paris Brain Institute—ICM, Inserm U1127, Paris 75013, France; Urgences cérébro-Vasculaires, Assistance Publique Hôpitaux de Paris, Sorbonne Université, Pitié-Salpêtrière Hospital, Paris 75013, France; Paris Brain Institute—ICM Stroke Network, STAR Team, Pitié-Salpêtrière Hospital, Paris 75013, France; CNRS UMR 7225, Sorbonne Université, Paris Brain Institute—ICM, Inserm U1127, Paris 75013, France; CNRS UMR 7225, Sorbonne Université, Paris Brain Institute—ICM, Inserm U1127, Paris 75013, France; INRIA, Aramis Project-Team, Paris 75013, France; CNRS UMR 7225, Sorbonne Université, Paris Brain Institute—ICM, Inserm U1127, Paris 75013, France; Service de Psychiatrie adulte, Assistance Publique Hôpitaux de Paris, Sorbonne Université, Pitié-Salpêtrière Hospital, Paris 75013, France; Service de Psychiatrie adulte, Assistance Publique Hôpitaux de Paris, Sorbonne Université, Pitié-Salpêtrière Hospital, Paris 75013, France; Institut Jean-Nicod, UMR 8129, ENS/EHESS/CNRS, IEC, PSL Research University, Paris 75005, France; Département de Neurologie, Assistance Publique Hôpitaux de Paris, Sorbonne Université, Pitié-Salpêtrière Hospital, Paris 75013, France; CNRS UMR 7225, Sorbonne Université, Paris Brain Institute—ICM, Inserm U1127, Paris 75013, France; Département de Neurologie, Assistance Publique Hôpitaux de Paris, Sorbonne Université, Pitié-Salpêtrière Hospital, Paris 75013, France; Département de Neurologie, Assistance Publique Hôpitaux de Paris, Sorbonne Université, Pitié-Salpêtrière Hospital, Institut de la Mémoire et de la maladie d’Alzheimer, Paris 75013, France; Département de Neurologie, Assistance Publique Hôpitaux de Paris, Sorbonne Université, Pitié-Salpêtrière Hospital, Unité d’épileptologie, Paris 75013, France; Département de Médecine Physique et de réadaptation, Assistance Publique Hôpitaux de Paris, Sorbonne Université, Pitié Salpêtrière Hospital, Paris 75013, France; Service de Neuro-orthopédie, Assistance Publique Hôpitaux de Paris, Sorbonne Université, Rothschild Hospital, Paris 75012, France; Département de Neurologie, Assistance Publique Hôpitaux de Paris, Sorbonne Université, Pitié-Salpêtrière Hospital, Paris 75013, France; Centre de Recherche en Myologie, UMRS974, Association Institut de Myologie, Sorbonne Université, Institut National de la Santé et de la Recherche Médicale, Paris 75013, France; Département de Neurologie, Assistance Publique Hôpitaux de Paris, Sorbonne Université, Pitié-Salpêtrière Hospital, Paris 75013, France; Service des Pathologies du sommeil, Assistance Publique Hôpitaux de Paris, Sorbonne Université, Pitié-Salpêtrière Hospital, Paris 75013, France; CNRS UMR 7225, Sorbonne Université, Paris Brain Institute—ICM, Inserm U1127, Paris 75013, France; Service de Psychiatrie adulte, Assistance Publique Hôpitaux de Paris, Sorbonne Université, Pitié-Salpêtrière Hospital, Paris 75013, France; Département de Biochimie Métabolique, Assistance Publique Hôpitaux de Paris, Sorbonne Université, Pitié-Salpêtrière Hospital, Paris 75013, France; CNRS UMR 7225, Sorbonne Université, Paris Brain Institute—ICM, Inserm U1127, Paris 75013, France; Département de Neuroradiologie, Assistance Publique Hôpitaux de Paris, Sorbonne Université, Pitié-Salpêtrière Hospital, Paris 75013, France; CNRS UMR 7225, Sorbonne Université, Paris Brain Institute—ICM, Inserm U1127, Paris 75013, France; Département de Neurologie, Assistance Publique Hôpitaux de Paris, Sorbonne Université, Pitié-Salpêtrière Hospital, Paris 75013, France; Département de Neurologie, Assistance Publique Hôpitaux de Paris, Sorbonne Université, Pitié-Salpêtrière Hospital, Unité d’épileptologie, Paris 75013, France; Département de Neurophysiologie Clinique, Assistance Publique Hôpitaux de Paris, Sorbonne Université, Pitié-Salpêtrière Hospital, Paris 75013, France; CNRS UMR 7225, Sorbonne Université, Paris Brain Institute—ICM, Inserm U1127, Paris 75013, France; Département de Neurologie, Assistance Publique Hôpitaux de Paris, Sorbonne Université, Pitié-Salpêtrière Hospital, Paris 75013, France; Département de Neurologie, Assistance Publique Hôpitaux de Paris, Sorbonne Université, Pitié-Salpêtrière Hospital, Paris 75013, France; Département de Neurologie, Assistance Publique Hôpitaux de Paris, Sorbonne Université, Pitié-Salpêtrière Hospital, Paris 75013, France; Département de Neurophysiologie Clinique, Assistance Publique Hôpitaux de Paris, Sorbonne Université, Pitié-Salpêtrière Hospital, Paris 75013, France; Unité de Soins intentifs neurovasculaires, Assistance Publique Hôpitaux de Paris, Sorbonne Université Saint-Antoine Hospital, Paris 75012, France; Département de Neurologie, Assistance Publique Hôpitaux de Paris, Sorbonne Université, Avicenne Hospital, Bobigny 93000, France; CNRS UMR 7225, Sorbonne Université, Paris Brain Institute—ICM, Inserm U1127, Paris 75013, France; Département de Neurologie, Assistance Publique Hôpitaux de Paris, Sorbonne Université, Pitié-Salpêtrière Hospital, Paris 75013, France; Service de Psychiatrie adulte, Assistance Publique Hôpitaux de Paris, Sorbonne Université, Pitié-Salpêtrière Hospital, Paris 75013, France; Département de Psychiatrie, Assistance Publique Hôpitaux de Paris, Sorbonne Université, Tenon Hospital, Paris 75020, France; Service de Gériatrie à orientation neurologique, Assistance Publique Hôpitaux de Paris, Sorbonne Université, Pitié-Salpêtrière Charles Foix Hospital, Paris 94200, France; Service de Gériatrie polyvalente, Assistance Publique Hôpitaux de Paris, Sorbonne Université, Charles Foix Hospital, Paris 94200, France; Service d’Addictologie, Assistance Publique Hôpitaux de Paris, Sorbonne Université, Pitié-Salpêtrière, Paris 75013, France; Service de Médecine Nucléaire et LIB, INSERM U1146, Assistance Publique Hôpitaux de Paris, Sorbonne Université, Pitié- Salpêtrière Hospital, Paris 75013, France; CNRS UMR 7225, Sorbonne Université, Paris Brain Institute—ICM, Inserm U1127, Paris 75013, France; Département de Neurologie, Assistance Publique Hôpitaux de Paris, Sorbonne Université, Pitié-Salpêtrière Hospital, Paris 75013, France; CNRS UMR 7225, Sorbonne Université, Paris Brain Institute—ICM, Inserm U1127, Paris 75013, France; CNRS UMR 7225, Sorbonne Université, Paris Brain Institute—ICM, Inserm U1127, Paris 75013, France; Département de Neurologie, Assistance Publique Hôpitaux de Paris, Sorbonne Université, Pitié-Salpêtrière Hospital, Paris 75013, France

**Keywords:** COVID-19, encephalopathy, encephalitis, critical illness neuropathy

## Abstract

A variety of neuropsychiatric complications has been described in association with COVID-19 infection. Large scale studies presenting a wider picture of these complications and their relative frequency are lacking. The objective of our study was to describe the spectrum of neurological and psychiatric complications in patients with COVID-19 seen in a multidisciplinary hospital centre over 6 months. We conducted a retrospective, observational study of all patients showing neurological or psychiatric symptoms in the context of COVID-19 seen in the medical and university neuroscience department of Assistance Publique Hopitaux de Paris—Sorbonne University. We collected demographic data, comorbidities, symptoms and severity of COVID-19 infection, neurological and psychiatric symptoms, neurological and psychiatric examination data and, when available, results from CSF analysis, MRI, EEG and EMG. A total of 249 COVID-19 patients with a *de novo* neurological or psychiatric manifestation were included in the database and 245 were included in the final analyses. One-hundred fourteen patients (47%) were admitted to the intensive care unit and 10 (4%) died. The most frequent neuropsychiatric complications diagnosed were encephalopathy (43%), critical illness polyneuropathy and myopathy (26%), isolated psychiatric disturbance (18%) and cerebrovascular disorders (16%). No patients showed CSF evidence of SARS-CoV-2. Encephalopathy was associated with older age and higher risk of death. Critical illness neuromyopathy was associated with an extended stay in the intensive care unit. The majority of these neuropsychiatric complications could be imputed to critical illness, intensive care and systemic inflammation, which contrasts with the paucity of more direct SARS-CoV-2-related complications or post-infection disorders.

## Teaser

Delorme et al. report neuropsychiatric manifestations in 245 COVID-19 patients hospitalized at the Pitié-Salpêtrière Hospital. The most frequent neuropsychiatric complications were encephalopathy, critical illness neuropathy, psychiatric disturbances and strokes, the majority being related to critical illness and intensive care, which contrasts with the paucity of more direct SARS-CoV-2-related complications.

## Graphical Abstract

**Graphical Abstract:**
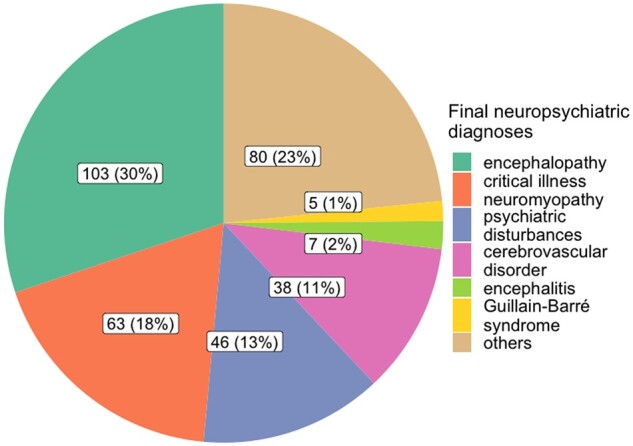

## Introduction

COVID-19, caused by the SARS-CoV-2 virus, has spread since December 2019 and was declared a pandemic by the World Health Organization. An initial Chinese cohort of 214 COVID-19 patients reported a high frequency of neurological symptoms (36%), including non-specific manifestations such as headache, confusion and myalgia, but also strokes and seizures.^1^ Since then, a variety of neurological complications have been described, including cerebrovascular complications,^2^ encephalopathy,^3^ encephalitis,^4^ seizures,^5^ Guillain–Barré syndrome,^6^ cranial nerve palsies,^7^ anosmia and dysgeusia.^8^ Several studies have raised concerns about a high prevalence of anxiety, mood disorders and post-traumatic stress disorders in COVID-19 patients.^9^^,^^10^ Besides case reports and case series illustrating the pleiotropy of COVID-19 neurological manifestations, cohort studies and registries have highlighted the particular high prevalence of strokes, encephalopathy and neuromuscular complications.^11–17^ More large-scale studies presenting a wide picture of these complications and of their relative frequency are needed.

We launched an observational study of the neuropsychiatric manifestations of COVID-19 at the onset of the first wave of the pandemic in France in March 2020. Here, we describe the detailed spectrum of neuropsychiatric disorders in 245 COVID-19 patients seen in the medical and university neuroscience department of APHP—Sorbonne University Hospitals over a 6-month period.

## Materials and methods

### Study design and population

This is a retrospective, observational study conducted consecutively on all COVID-19 patients with neurological or psychiatric symptoms seen between March 1st and August 28th at the *APHP* – *Sorbonne University* medical and university neuroscience department, which groups the neurology and psychiatry departments of five Hospitals in Paris (Pitié-Salpêtrière, Saint-Antoine, Tenon, Rothschild, Charles-Foix hospitals). The study Investigators were physicians from the department, which includes all medical units in the field of adult neurology, neurovascular, neurological intensive care, neurorehabilitation, psychiatry, neurophysiology and neuropathology.

All consecutive in- or out-patients, aged 18 years or older, with COVID-19 and *de novo* neurological or psychiatric symptoms were included in the study. We included patients hospitalized or seen as outpatients in the neuroscience department, but also patients reported by physicians working in other departments involved in the care of COVID-19 patients after informing all physicians of our university hospital that patients with neurological or psychiatric symptoms should be reported. Patients showing uniquely anosmia and/or dysgeusia were not included. Patients presenting with acute exacerbations of known pre-existing neurological disorders were not included. We included patients with pre-existing psychiatric disorders only if they presented with acute decompensations with clear disruption in their clinical course.

The primary objective of the study was to describe the spectrum of neurological and psychiatric complications in COVID-19 patients.

### Data collection

Data were collected retrospectively by investigators from medical records and entered into a structured case report form. Items collected included demographic data, medical and treatment history, comorbidities, symptoms, date of onset, and severity of COVID-19 infection, neurological and psychiatric symptoms, neurological and psychiatric examination, and, when available, results from CSF analysis, brain imaging including MRI, EEG and electroneuromyography (ENMG).

COVID-19 was defined by at least one of the three following criteria: (i) positive SARS-CoV-2 polymerase chain reaction in swab or upper pulmonary samples, or positive serology; (ii) thoracic radiological findings typical of SARS-CoV2 infection; and (iii) suspected COVID-19 infection according to the World Health Organization guidance criteria.^18^ The severity of COVID-19 was the status of the patient at the nadir of the disease according to the seven levels as defined by the World Health Organization: 1-not hospitalized, no limitation in daily life activity; 2-not hospitalized, with limitation in daily life activity; 3-hospitalized, no oxygen requirement; 4-hospitalized, necessitating oxygen; 5-hospitalized, necessitating non-invasive ventilation or Optiflow™; 6-hospitalized, necessitating intubation or extracorporeal membrane oxygenation; and 7-death.

The investigators completed the database consisting of a predefined list of neurological and psychiatric characteristics (symptoms, clinical signs, date of onset). Each item was scored as present, absent or unknown (no clinical evaluation available), and a final neurological or psychiatric syndrome was determined. One investigator (C.D.) reviewed all cases, and classified the diagnoses according to criteria of the Liverpool Brain infections Group (Neuro Network).^19^ We used the word ‘encephalopathy’ to refer to acute global disturbances in cognition (encompassing delirium, confusional states and altered mental status).^20^ When a patient had more than one diagnosis, the diagnoses were classified as primary (pronounced) or secondary.

### Standard protocol approvals

The study was conducted in accordance with good clinical practice, the French regulation for retrospective studies on clinical data, and was compliant with the European General Data Protection Regulation (GDPR) and the French *Commission Nationale de l’Informatique et des Libertés* (CNIL) rules. All patients (or their relatives in cases of impaired consciousness) received written information about the research, and consented to the use of their data. The study received the approval of the Sorbonne University Ethics Committee (N°2020 CER-202028). The study is registered on the clinicaltrials.gov website (NCT04362930).

### Statistical analysis

For the analyses, we grouped the 7-level COVID-19 severity score into four categories: 1-not hospitalized (levels 1 and 2); 2-hospitalized without intensive care (levels 3 and 4); 3-hospitalized with intensive care (levels 5 and 6); and 4-death (level 7). Kruskal–Wallis tests and Fisher’s Exact tests were used to compare groups. Pairwise comparisons were performed using pairwise Wilcoxon–Mann–Whitney tests and pairwise Fisher’s Exact tests with Benjamini–Hochberg method to correct for multiple testing.

We performed adjacent category ordinal logistic regression on the four grouped categories of the 7-levels score with the risk factors age, gender, obesity (body mass index ≥30 kg/m^2^) or other comorbidity.

Neuropsychiatric symptoms, signs and syndromes were assessed on non-missing data.

For the correlation matrix, we calculated the Pearson correlation for inter-relationships between the following variables: age, gender, presence of one comorbidity, intensive care unit (ICU) hospitalization and the various neurological syndromes. Hierarchical clustering was applied with the single linkage distance.

The risk factors for the most frequent neurological syndromes were analysed using the Classification And Regression Tree algorithm. The Classification And Regression Tree algorithm, also known as a ‘decision tree’, is a non-parametric supervised technique that combines variables in such a way as to best discriminate two groups. For each neurological syndrome, we trained one tree to depth 4 through entropy minimization.

Statistical analyses were performed using R 3.6.1. and package VGAM_(version 1.1–3) for the ordinal logistic regression (R Foundation for Statistical Computing, Vienna, Austria. URL https://www.R-project.org/.) and using python 3.8 with the scikit learn 0.23.2 package for decision trees and correlation matrices.^21^

### Data availability

All data are available upon request to the corresponding author.

## Results

### Patient characteristics

During the study period, 1979 patients were admitted with a diagnosis of COVID-19 in our centre. A total of 249 COVID-19 patients with a *de novo* neurological or psychiatric manifestation were included in the database. Of these 249 patients, 24 were seen as outpatients and 225 were hospitalized (11.3% of all hospitalized COVID-19 patients). Three patients were excluded because they withdrew their consent, one patient was excluded because he did not fulfil the diagnosis criteria for COVID-19. The population analysed consisted of 245 cases.

The characteristics of the patients are presented in Table 1.

**Table 1:** Comparison between COVID-19 severity groups

	All *N* = 245	1. Non-hospitalized (a) *N* = 24 (9.8%)	2. Hospitalized (b) *N* = 96 (39.2%)	3. ICU (c) *N* = 114 (46.5%)	4. Deaths (d) *N* = 10 (4.1%)	*P*‡
Age	63.8 [50.2, 72.7]	42.2 [32.3, 55.9] b, c, d	72.1 [62.2, 85.9] a, c	59.4 [50.2, 66.9] a, b, d	74.1 [65.5, 77.0] a, c	<0.001*
Age < 40	29 (12%)	11 (48%) b, c, d	6 (6%) a	12 (11%) a	0 (0%) a	<0.001*
Age > 60	140 (58%)	5 (22%) b, d	72 (76%) a, c	54 (47%) b	8 (80%) a	<0.001*
Gender (F)	97 (40%)	11 (46%)	48 (50%) c	34 (30%) b	4 (40%)	0.020*
Ethnicity						0.017*
Caucasian						0.008*
No	96 (39%)	5 (21%) c	33 (34%) c	53 (46%) a, b	5 (50%)	
Yes	92 (38%)	13 (54%)	46 (48%)	29 (25%)	3 (30%)	
Not available	57 (23%)	6 (25%)	17 (18%)	32 (28%)	2 (20%)	
Comorbidities	190 (78%)	9 (39%) b, c	73 (76%) a	99 (88%) a	8 (80%)	<0.001*
Cardiac disease	131 (54%)	2 (9%) b, c, d	55 (57%) a	68 (60%) a	6 (60%) a	<0.001*
Respiratory disease	26 (11%)	2 (9%)	16 (17%)	7 (6%)	1 (10%)	0.089
Diabetes	70 (29%)	2 (9%)	25 (26%)	39 (35%)	4 (40%)	0.045*
Active smoking	39 (16%)	3 (13%)	12 (12%)	22 (19%)	2 (20%)	0.528
Obesity (BMI > 30 kg/m²)	52 (21%)	2 (9%)	12 (12%) c	37 (33%) b	1 (10%)	<0.001*
Immunodeficiency	15 (6%)	0 (0%)	5 (5%)	9 (8%)	1 (10%)	0.401
Cancer	11 (5%)	0 (0%)	10 (10%) c	1 (1%) b	0 (0%)	0.008*
Other comorbidity	22 (9%)	1 (4%)	9 (9%)	9 (8%)	2 (20%)	0.481
COVID-19 diagnosis by PCR	204 (89%)	14 (74%)	81 (88%)	98 (92%)	10 (100%)	0.121
COVID-19 diagnosis by CT	164 (81%)	8 (67%)	62 (77%)	85 (86%)	8 (89%)	0.180
Clinical severity						NA
1-Not hospitalized, no limitation in daily life activities	11 (5%)	11 (46%)				
2-Not hospitalized, with limitation in daily life activity	13 (5%)	13 (54%)				
3-Hospitalized, no need of oxygen	46 (19%)		46 (48%)			
4-Hospitalized, necessitate oxygen	50 (20%)		50 (52%)			
5-Hospitalized necessitate non-invasive ventilation or optiflow	11 (5%)			11 (10%)		
6-Hospitalized, necessitate intubation or ECMO	103 (42%)			103 (90%)		
7-Death	10 (4%)				10 (100%)	
COVID-19 symptoms						
Fever	183 (76%)	18 (75%)	69 (73%)	90 (81%)	6 (60%)	0.301
Cough	149 (63%)	12 (50%)	52 (55%)	79 (73%)	6 (67%)	0.028*
Chest pain	26 (12%)	5 (21%)	7 (8%)	13 (13%)	1 (14%)	0.230
Myalgia	45 (20%)	11 (46%) b, c	15 (17%) a	17 (17%) a	2 (29%)	0.014*
Dyspnea	141 (60%)	6 (25%) c, d	42 (45%) c, d	83 (77%) a, b	10 (100%) a, b	<0.001*
Nausea/Vomitting	34 (15%)	5 (21%)	13 (14%)	15 (14%)	1 (12%)	0.865
Diarrhea	52 (23%)	6 (25%)	17 (19%)	27 (26%)	2 (25%)	0.659
Fatigue	115 (50%)	17 (71%)	52 (55%)	42 (40%)	4 (44%)	0.021*
Anosmia	39 (18%)	11 (48%) b, c	10 (11%) a	17 (17%) a	1 (17%)	<0.001*
Dysgueusia	28 (13%)	9 (39%) b, c	5 (6%) a	13 (13%) a	1 (14%)	<0.001*

Data are given as median [first, third quartiles] for continuous variables and as count (percentages) for categorical variables.

‡ Kruskal–Wallis test was used to compare groups for continuous variables and Fisher’s exact test for qualitative variables. Pairwise Wilcoxon–Mann–Whitney tests for continuous variables and pairwise Fisher’s exact tests for qualitative variables were performed using Benjamini–Hochberg method to correct for multiple testing. Following letters indicate which groups significantly differ: a group differs from 1. non-hospitalized; b group differs from 2. hospitalized; c group differs from 3. ICU; d group differs from 4. deaths.

BMI, body mass index; CT, computed tomography; ECMO, extracorporeal membrane oxygenation; F, female; ICU, intensive care unit; *N*, number; NA, not applicable; PCR, polymerase chain reaction.

The most frequent symptoms of COVID-19 were fever (76%), cough (63%), dyspnoea (60%) and fatigue (50%). One-hundred and fourteen patients (47%) were admitted to the ICU with a median stay of 27 days, and 10 (4%) died. Male gender, non-Caucasian origin and the presence of comorbidities (obesity, diabetes, cardiopathy, cancer) were associated with a greater COVID-19 severity. Older age was associated with greater COVID-19 severity but not with ICU admission (Tables 1 and 2).

**Table 2:** Results from adjacent category logit model on COVID-19 severity

	P [*Y* = 2. hospitalized]/ P [*Y* = 1. non-hospitalized]		P [*Y* = 3. ICU]/ P [*Y* = 2. hospitalized]		P [*Y* = 4. death]/ P [*Y* = 3. ICU]	
	OR [CI 95%]	*P*	OR [CI 95%]	*P*	OR [CI 95%]	*P*
Age	1.12 [1.07–1.16]	<0.001*	0.95 [0.93–0.97]	<0.001*	1.06 [1.01–1.12]	0.018*
Gender (M)	1 [0.32–3.13]	0.998	2 [1.05–3.81]	0.034*	0.81 [0.19–3.44]	0.772
At least one comorbidity (yes)	3.5 [1.05–11.62]	0.041*	1.96 [0.86–4.50]	0.110	0.63 [0.11–3.50]	0.598
Obesity (yes)	1.41 [0.23–8.53]	0.707	2.37 [1.05–5.35]	0.037*	0.36 [0.04–3.28]	0.367

For categorical effects, category in brackets is not the reference.

CI, confidences intervals; ICU, intensive care unit; M, male; OR, odds ratios.

### Neurological and psychiatric symptoms and signs

**Figure 1:**
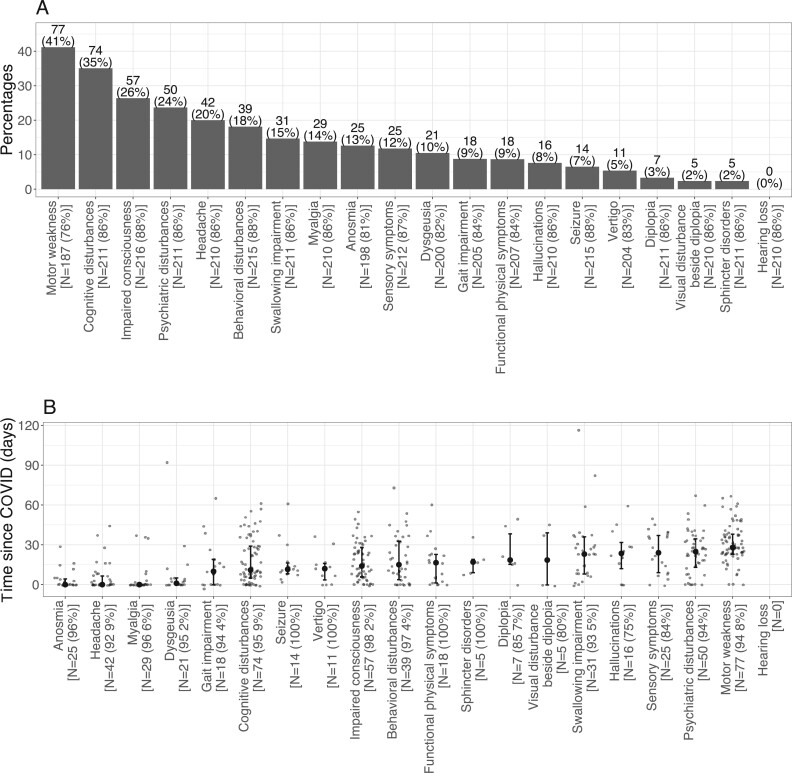
**Neuropsychiatric symptoms and their delays since Covid-19 onset.** (**A**) Neuropsychiatric symptoms repartition. For each symptom, the number and percentage of non-missing patients is given below. (**B**) Delay between symptom and COVID onset for each symptom. Median, first and third quartiles are represented. The number of subjects with the symptom as well as the percentage of available delays among them are given below.

Neuropsychiatric symptoms were very diverse (Fig. 1A). The most frequently reported were motor weakness (41%), cognitive disturbances (35%), impaired consciousness (26%), psychiatric disturbance (24%), headache (20%) and behavioural disturbance (18%).

The delay between COVID-19 onset and neuropsychiatric symptoms ranged from 0 to 116 days (Fig. 1B). Some symptoms appeared soon after COVID-19 onset: myalgia (median 0 days), headache (0 days), anosmia (0 days), dysgeusia (1 day), gait impairment (10 days), while others appeared with a delayed onset, such as sensory symptoms (24 days), psychiatric disturbances (25 days) or motor weakness (28 days).

The most frequent clinical signs were motor weakness (41%) and cognitive disurbance (38%) [temporo-spatial disorientation (33% of the total population), memory disturbances (26%), language disorders (18%), frontal syndrome (14%)]. Cranial nerve examination was abnormal in 21% of the patients (cranial nerve II: 2 patients, III, IV or VI: 15 patients, V: 4 patients, VII: 16 patients, VIII: 2 patients, IX or X: 12, XI: 4 patients, XII: 5 patients), either seen in the context of brainstem global dysfunction or isolated cranial nerve palsies. Movement disorders, mostly myoclonus and myoclonic tremor, were seen in 14% of the patients and cerebellar syndrome in 2%. Pyramidal syndrome was present in only 12% of the patients and areflexia in 10%.

### Paraclinical explorations

CSF was collected in 53 (22%) patients. Only six patients (11%) showed evidence of CSF hypercellularity (leucocytes >5/mm^3^), ranging from 6 to 205 leucocytes/mm^3^. Protein count was above 0.40 g/l in 17 patients (0.42–2.9 g/l). SARS-CoV-2 polymerase chain reaction was negative in the CSF for all patients in which this analysis was performed (38 patients).

Brain MRI was performed in 119 patients and was abnormal in 81 (68%, 9 patients with missing data). MRI findings comprised ischaemic strokes, intracerebral haemorrhages, cerebral venous thrombosis, cytotoxic lesions, basal ganglia abnormalities and white matter enhancing lesions. They have been described elsewhere.^22^

EEG was performed in 82 patients and was abnormal in 54 patients (77%, 12 with missing data). Detailed EEG data are presented in another paper.^23^ Pathological EEG findings included focal abnormalities, metabolic-toxic encephalopathy, periodic discharge and epileptic activity.

ENMG was carried out in 25 patients and was abnormal in 20 patients with evidence of peripheral nervous system impairment (87%, 2 patients with missing results).

### Syndromes and causes of neuropsychiatric complications

**Figure 2:**
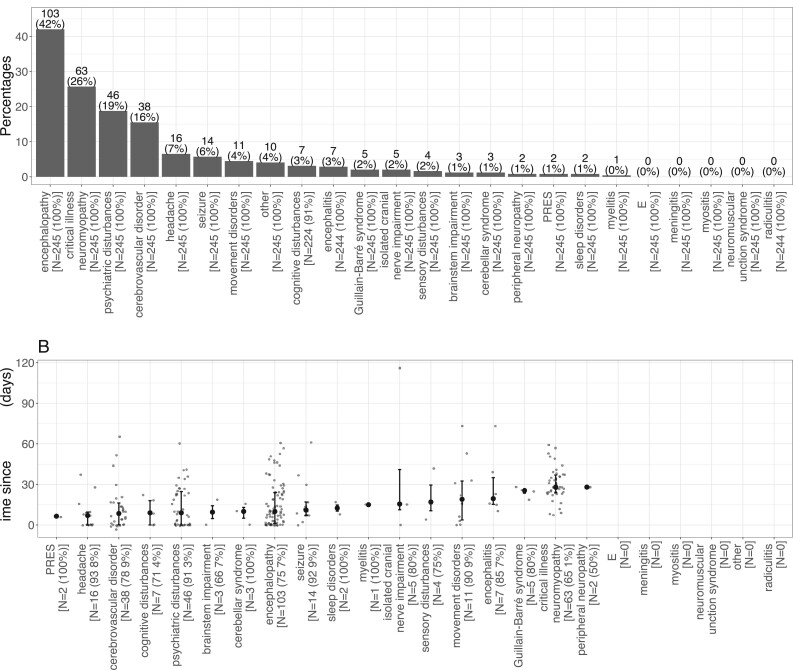
**Final neuropsychiatric diagnoses and their delays since Covid-19 onset.** (**A**) Neuropsychiatric syndromes repartition. For each syndrome, the number and percentage of non-missing patients is given below. (**B**) Delay between syndrome and COVID onset for each syndrome. Median, first and third quartiles are represented. The number of subjects with the syndrome as well as the percentage of available delays among them are given below.

The most frequent neuropsychiatric syndromes were encephalopathy (42%), critical illness polyneuropathy and myopathy (26%), isolated psychiatric disturbance (18%) and cerebrovascular disorders (16%) (Fig. 2A). Other syndromes were much rarer: isolated disabling headache (7%), seizures (6%), isolated movement disorders (4%), cognitive disturbance without encephalopathy (3%) and encephalitis (3%). Guillain–Barré syndrome was observed in five patients, and isolated cranial nerve impairment in five patients. Two patients had posterior reversible encephalopathy syndrome. One patient had cervical myelitis confirmed on spine MRI. Three patients displayed a cerebellar syndrome. Three patients had signs of brainstem impairment. Four patients complained of subjective sensory signs without ENMG abnormalities.

The delay between COVID-19 onset and each neuropsychiatric syndrome onset ranged from 0 to 116 days (Fig. 2B). Cerebrovascular disorders, cognitive disturbance, headache and psychiatric disturbance usually occurred within the first 10 days following COVID-19 onset. Conversely, myelitis, encephalitis and cranial nerve palsies occurred around 15 days, Guillain–Barré around 25 days, and critical-illness neuromyopathy after 28 days from COVID-19 onset.

#### Encephalopathy

Among patients with encephalopathy, 56% were hospitalized in the ICU. Twenty per cent of patients with encephalopathy had a pre-existing cognitive disorder. Seventy per cent of the patients with encephalopathy had at least one cardiological, respiratory or metabolic comorbidity. The most common clinical presentations of encephalopathy were confusion, and delayed awakening after stopping sedative drugs. Among the ten deceased patients, seven (70%) showed encephalopathy.

With respect to risk ratios, encephalopathy mainly affected patients over 60 years of age (Table 3).

**Table 3:** Risk ratios for each potential risk factor of the 3 most frequent syndromes.

Potential risk factors	Critical illness neuromyopathy *N* = 63	Cerebrovascular disorder *N* = 38	Encephalopathy *N* = 106
Gender (female) (*n* = 97)	0.61 (*n* = 18)	0.70 (*n* = 12)	**0.78 (*n* = 36)**
No comorbidities (*n* = 58)	0.47 (*n* = 8)	**0.60 (*n* = 6)**	**0.66 (*n* = 18)**
Comorbidity			
Cardiac disorder (*n* = 131)	0.96 (*n* = 33)	**2.44 (*n* = 28)**	1.18 (*n* = 61)
Obesity (*n* = 52)	2.13 (*n* = 23)	0.84 (*n* = 7)	0.81 (*n* = 19)
Diabetes (*n* = 70)	1.44 (*n* = 23)	1.30 (*n* = 13)	1.29 (*n* = 36)
Age			
Age < 40 (*n* = 31)	1.00 (*n* = 8)	0.81 (*n* = 4)	0.20 (*n* = 3)
40 < age < 60 (*n* = 74)	1.73 (*n* = 27)	1.51 (*n* = 15)	0.96 (*n* = 31)
60 < age < 80 (*n* = 105)	1.07 (*n* = 28)	0.87 (*n* = 15)	1.33 (*n* = 31)
80 < age (*n* = 35)	0.00 (*n* = 0)	0.71 (*n* = 4)	1.31 (*n* = 19)
ICU (*N* = 114)	**22.98 (*n* = 60)**	0.41 (*n* = 10)	1.29 (*n* = 56)

In bold, the significant risk factors.

ICU, intensive care unit.

**Figure 3:**
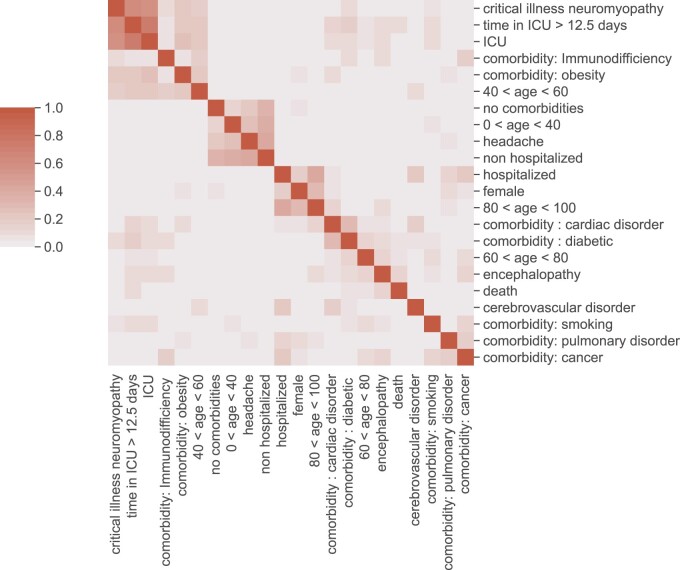
Statistical associations between neuropsychiatric syndromes and risk factors. Correlation matrix between neuropsychiatric syndromes, comorbidities and other related variables. Shades of red indicates a positive correlation. The variables were clustered by minimizing the single linkage distance.

In the correlation matrix, encephalopathy was associated with higher age, death, cardiac and diabetic comorbidities (Fig. 3).

#### Critical illness polyneuropathy and myopathy

Critical illness neuropathy or myopathy was diagnosed in the recovery phase after sedative drug reduction in the ICU. ENMG findings were available for 22% of these patients. Several patterns of neuromuscular injury were observed: (i) axonal sensorimotor polyneuropathy, (ii) myopathy, (iii) troncular nerve compressions (median, ulnar, peroneal, lateral femoral cutaneous nerves), and (iv) brachial plexopathy. Brachial plexopathy was only seen in patients after remaining prone for extended periods. Thirty-seven percent of the patients with critical illness polyneuropathy had pre-existing diabetes.

The decision tree showed that a stay exceeding 12.5 days was the strongest feature predictive of a neuropathy after entering the ICU (Supplementary Fig. 1).

#### Psychiatric disorders

Among the 28 patients with available details for their psychiatric disturbances, 71% had a pre-existing psychiatric disorder: depression (70%), anxiety disorders (20%), psychosis (20%), bipolar disorder (10%), substance abuse disorder (5%).

The most commonly observed psychiatric disorders in the context of COVID-19 infection were anxiety disorders (25%), depression (18%), acute psychosis (20%) (of which 2/5 patients had pre-existing psychosis), adjustment disorders (7%) and acute stress (3%).

#### Cerebrovascular disorders

Thirty-eight patients (16%, median [Q1, Q3] age 62.3 [52.1,70.1]; 26 males, 68%) suffered strokes with the following proportions: 32 (84%) ischaemic strokes (regional or multiple small infarcts), three (8%) parenchymal haematomas, one subarachnoid haemorrhage and one cerebral venous thrombosis. Patients with cerebrovascular disorders had the following cardiovascular risk factors: hypertension (22, 58%), diabetes mellitus (13, 34%), dyslipidemia (9, 24%) and obesity (7, 18%). The correlation matrix indeed showed the association between cerebrovascular disorders and comorbidities (smoking, pulmonary disorder, cancer) (Fig. 3). Seven patients had a history of previous stroke.

Unusual symptoms at presentation (which were not explained by infarct location or metabolic disturbances) were frequently found such as confusion (13/32, 40%) or apathy (5/38, 13%).

A fraction of these patients has been already reported.^24^

#### Seizures

Among the 14 patients with seizures, none had a previous history of epilepsy. One patient was under treatment for Parkinson’s disease with dementia and one patient for glioblastoma. Three patients had a focal seizure without generalization, one patient a focal to bilateral generalized seizure, seven patients a generalized seizure, one patient a status epilepticus.

#### Cranial nerve palsies

Five patients presented with cranial nerve palsies. One patient had VI nerve palsy with normal MRI and CSF examination. As she was a heavy smoker, a thrombosis triggered by the COVID-19 infection was suspected. One patient presented with optic neuritis, in the setting of a possible inflammatory disorder of the CNS (inflammatory lesions on brain MRI, oligoclonal bands in the CSF). One patient presented with III nerve partial palsy after an ICU stay. One patient presented with unilateral hypoglossal nerve palsy, and one with combined homolateral X, XI and XII nerves palsies, while in the ICU, which were attributed to mechanical complications of positioning, intubation or jugular catheterizations.

#### Headaches

Isolated disabling primary headache was the primary diagnosis in 16 patients (7%). Headaches often had migraine characteristics. None of the patients had pre-existing migraines. Among patients with headache, eight underwent brain MRI, which was normal in seven patients and showed a non-specific lesion in one. Two had lumbar puncture (normal in both).

In the correlation matrix, headache is associated with younger patients who were not hospitalized and showed no associated comorbidities (Fig. 3).

#### Encephalitis

Only seven patients fulfilled criteria for encephalitis. Among those seven patients, one patient had positive polymerase chain reaction for varicella-zoster-virus in the CSF, suggesting concomitant varicella-zoster-virus encephalitis. One patient had an encephalopathy with myoclonus and inflammatory brain lesions, one patient had cognitive disturbances with myoclonus and CSF pleiocytosis, one patient had brainstem impairment, movement disorders and dysautonomia with white matter lesions on MRI, two patients showed alterations of consciousness with white matter lesions on MRI, one patient had confusion with MRI features of limbic encephalitis.

Several of these patients have been already reported.^25^

#### Guillain–Barré syndrome

Five patients were diagnosed with Guillain–Barré syndrome, three of them requiring hospitalization in the ICU. One patient had a pre-existing demyelinating Charcot–Marie–Tooth disease. In four patients, Guillain–Barré syndrome presented with severe motor weakness of all four limbs. One of the patients had associated bilateral facial nerve palsy. CSF showed elevated protein in two patients (1.1 g/l and 1.65 g/l). ENMG showed typical characteristics of acute inflammatory demyelinating polyneuropathy in all of them.

## Discussion

We describe a wide range of neuropsychiatric symptoms and syndromes occurring in COVID-19 patients seen in a single multidisciplinary centre over a 6-month period. Our patient cohort primarily presented with encephalopathy, critical illness polyneuropathy and myopathy, psychiatric disturbances, and cerebrovascular complications. The prevalence of neuropsychiatric symptoms among patients hospitalized in our COVID-19 centre was 11%. The prevalence of neurological signs reported in previous studies has been very variable, depending on inclusion criteria and evaluation methods, varying between 4.2%^26^ and 57.4%.^27^ The relatively lower prevalence in our study may be due to the strict inclusion criteria and the exclusion of patients with isolated symptoms of anosmia or dysgeusia. Compared to those with non-severe disease, the patients in ICU were more likely to have comorbidities, including hypertension, diabetes, cancer, cardiac or kidney disease.

Our cohort highlights the high frequency of neuropsychiatric complications related to critical illness and intensive care. Almost half of the patients in our cohort were hospitalized in the ICU (47%). Our findings emphasize that encephalopathy is a major issue in patients with COVID-19. This has been previously reported by previous teams which variably referred to encephalopathy,^28^ delirium^29^ and altered mental status,^30^ which we chose to regroup under the term encephalopathy.^20^ The presence of encephalopathy was strongly associated with COVID-19 infection severity and the presence of comorbidities, which is in keeping with complications of hypoxia and critical illness. The high prevalence of encephalopathy in patients with COVID-19 patients has already been reported, and seems to be more common in patients with more severe COVID-19-related respiratory disease, associated comorbidities, evidence of multi-organ system dysfunction, including hypoxaemia, renal and hepatic impairment, and elevated markers of systemic inflammation.^1^^,^^31^^,^^32^ The association of encephalopathy with greater age and comorbidities has already been reported.^33^^,^^34^ A recent large-scale cohort also emphasized the frequency of non-specific complications, including toxic/metabolic encephalopathy and hypoxic/ischaemic brain injury.^13^ The prevalence of encephalopathy is very high in patients with COVID-19 hospitalized in the ICU.^35^ As in previous works, we found that encephalopathy was associated with the risk of death.^34^^,^^36^^,^^37^

Critical care polyneuropathy and myopathy were particularly common in our patients. Besides classical ICU polyneuropathy, many patients presented with mechanic plexopathy and nerve compressions, probably secondary to prolonged sedation and lying prone. The occurrence of critical illness polyneuropathy and myopathy was statistically associated with a longer stay in the ICU. The long duration of critical care, the requirement for high doses of anaesthetics and curare, and the frequent association with diabetes could partly explain the particularly high frequency of ICU neuropathy. Two patients presented with mechanical cranial nerve injuries (XII in one patient; X, XI and XII in one patient) in the setting of critical care, a complication known as Tapia syndrome.^38^

Acute cerebrovascular disorders consisted mostly in ischaemic strokes. The risk of cerebrovascular disorders was highly associated with the presence of comorbidities. The presence of unusual stroke symptoms at presentation, such as encephalopathy without focal deficit, is noteworthy and was also reported by other authors.^39^ COVID-19 might facilitate ischaemic strokes via at least three non-exclusive pathogenic mechanisms: atherosclerotic plaque vulnerability, a hypercoagulable state, and cerebral microvasculature injury (endothelitis).^40–42^ Indeed, COVID-19 is commonly complicated by sepsis-induced coagulopathy, induced by a systemic inflammatory response involving endothelial dysfunction and microthrombosis often associated with multi organ failure.^43^

These observations contrast with the rarity of syndromes potentially linked to viral neuroinvasion. The question of the neuroinvasiveness of the SARS-CoV-2 is an ongoing debate. Its detection in the CSF has only been reported in a few case studies.^4^^,^^44^ The majority of neuropathological observations favour the role of critical illness complications or described non-specific inflammatory brain lesions,^45–47^ although some recent findings have shed new light on the neuroinvasive potential of SARS-CoV-2.^41^^,^^48^ A recent cohort with 606 patients with neurological signs of COVID-19 reported no encephalitis.^13^ Only seven patients in our cohort fulfilled the diagnostic criteria for encephalitis, and none had evidence of SARS-CoV-2 detection in the CSF.^19^ The CSF pleiocytosis without SARS-CoV-2 detection and brain inflammatory lesions seen in our patients and other reported cases of COVID-19 might result from immune-mediated inflammatory mechanisms rather than direct viral invasion. The role of cytokine activation has been widely reported for COVID-19.^49^,^50^ Headaches are also proposed to be secondary to inflammatory cytokinic mechanisms,^51^ which may also play and important role in psychiatric manifestations.^52^ Other immune-mediated complications, such as Guillain–Barré syndrome and myelitis occurred anecdotally in our cohort. Cranial nerve impairments could often be explained by alternative mechanisms (pre-existing inflammatory disorder, thrombotic mechanism, mechanical compressions). 

Although our sample of patients who developed psychiatric symptoms during COVID-19 is small, our findings are in accordance with previously reported data.^53^ The occurrence of psychiatric symptoms in patients with COVID-19 was remarkably high, which is in keeping with previous studies showing that psychiatric symptoms are more frequent in patients with COVID-19 than with other infections or health events.^54^ Indeed, our patients mainly suffered from anxiety disorder or depression, and none developed obsessive compulsive disorder or bipolar disorder. Five patients presented with acute psychosis. The difficulty to distinguish acute psychosis and delirium has been highlighted by some authors.^55^ In our multidisciplinary centre, each patient suspected of experiencing a psychotic episode was evaluated by a psychiatrist at several time points; thus, we paid a special attention to know if the patient met the DSM-5 criteria of delirium (disturbance in attention and awareness; disturbance develops over a short period of time and tends to fluctuate in severity during the course of a day; an additional disturbance in cognition) or the criteria for an acute psychotic episode. Long-term follow-up data will be valuable to have an estimation of the risk of developing post-traumatic stress disorder, especially for patients with adjustment disorders and acute stress syndrome.

Our study has several limitations. Although the study is retrospective, it was implemented early at the onset of the COVID-19 pandemic and data could be collected prospectively by the investigators as they examined new patients. Furthermore, the absence of a control group of patients with COVID-19 without neurological or psychiatric manifestations limits the overall interpretation of the results. We may have an over representation of complications due to critical illness and critical care as there was a large number of COVID-19-dedicated ICU beds in our institution. We cannot exclude reporting bias and lack of exhaustivity, but the involvement of all physicians of the Department implicated in every step of patient care (ICU, acute hospitalization departments, rehabilitation departments, imaging departments, neurophysiology) limits this bias, especially for the most severe forms. In stroke patients, one limitation of the study is that we could not determine for all patients the aetiology of ischaemic strokes and in case of a negative outcome, whether COVID-19 was the trigger.

In conclusion, we report the broad landscape of neuropsychiatric complications in a large cohort of COVID-19 patients. The majority of these complications could be attributed to critical illness, intensive care and systemic inflammation, which contrasts with the paucity of more direct SARS-CoV2-related complications or post-infectious disorders. Further studies are needed to better disentangle the different mechanisms underlying these various symptoms, and to explore potential long-term complications.

## Supplementary material

Supplementary material is available at *Brain Communications* online.

## Supplementary Material

fcab135_Supplementary_Data

## Abbreviations

ENMG =: electroneuromyography
ICU =: intensive care unit
