# Supplementary material for: Spectrum, risk factors and outcomes of neurological and psychiatric complications of COVID-19: a UK-wide cross-sectional surveillance study
Source: Brain Commun. Author manuscript; Available in PMC 2021 Aug 10. (PMC8344449; doi:10.1093/braincomms/fcab135)
Supplement: Supplementary [file EMS133140-supplement-Supplementary.docx]

**SUPPLEMENTARY DATA FOR:**

Spectrum, risk factors, and outcomes of neurological and psychiatric complications of COVID-19: a UK-wide cross-sectional surveillance study

Amy L Ross Russell, Marc Hardwick, Athavan Jeyanantham, Laura M White, Saumitro Deb, Girvan Burnside, Harriet M Joy, Craig J Smith, Thomas A Pollak, Timothy R Nicholson, Nicholas WS Davies, Hadi Manji, Ava Easton, Stephen Ray, Michael S Zandi, Jonathan P Coles, David K Menon, Aravinthan Varatharaj, Beth McCausland, Mark A Ellul, Naomi Thomas, Gerome Breen, Stephen Keddie, Michael P Lunn, John PS Burn, Graziella Quattrocchi, Luke Dixon, Claire M Rice, George Pengas, Rustam Al-Shahi Salman, Alan Carson, Eileen M Joyce, Martin R Turner, Laura A Benjamin, Tom Solomon, Rachel Kneen, Sarah Pett, Rhys H Thomas, Benedict D Michael, Ian Galea, on behalf of the CoroNerve Studies Group

**LIST OF COMORBIDITIES**

Ischaemic heart disease

Heart failure or dilated cardiomyopathy

Atrial fibrillation

Hypertension

Valvular heart disease: any type

Takayasu's disease

Congenital heart disease: tetralogy of Fallot

Peripheral vascular disease

Neurodegenerative disease: any type

Previous subdural haematoma

Epilepsy

Cerebrovascular disease including previous transient ischaemic attacks

CNS vasculitis

Brain tumour

Multiple sclerosis

Previous deep vein thrombosis

Previous pulmonary embolism

Osteoporosis

Chronic obstructive pulmonary disease

Obesity related hypoventilation syndrome

Obstructive sleep apnoea

Bronchiectasis

Asthma

Pulmonary hypertension

Previous pulmonary tuberculosis

Hypothyroidism

Chronic kidney disease: diabetic nephropathy, hypertensive, unspecified, anti-glomerular basement membrane disease, glomerulonephritis

Hydronephrosis

Renal transplant

Polycystic kidney disease

CADASIL (Cerebral Autosomal Dominant Arteriopathy with Subcortical Infarcts and Leukoencephalopathy)

Human immunodeficiency virus

Latent syphilis

Chronic hepatitis C

Mannan-binding lectin deficiency

Previous gastric ulcer or upper GI bleed

Oesophagitis, gastritis and duodenitis

Inflammatory bowel disease: Crohn's, ulcerative colitis, coeliac

Oesophageal stricture with dysplasia: likely active cancer

Alcoholism

Type 1 and type 2 diabetes mellitus

Pernicious anaemia

Sickle cell disease

Any haematological malignancy

Obesity

Gout

Rheumatoid arthritis

**LIST OF NON-NEUROLOGICAL THROMBOTIC EVENTS**

Pulmonary embolism

Deep vein thrombosis

Left ventricular thrombus

Myocardial infarction

Renal artery thrombus

Right axillary vein thrombus

Jugular vein thrombosis

Disseminated intravascular coagulation

**SUPPLEMENTARY TABLES**

| **Diagnostic group** | **Specific diagnosis** | **Number of cases** | **Reference** |
| --- | --- | --- | --- |
| Central inflammatory - Demyelination/leukoencephalopathy | CNS inflammatory vasculopathy with antimyelin oligodendrocyte glycoprotein antibodies | 1 | <https://doi.org/10.1212/NXI.0000000000000813> |
| Central inflammatory - Demyelination/leukoencephalopathy | Acute disseminated encephalomyelitis with corpus callosal haemorrhage | 1 | <https://doi.org/10.1212/WNL.0000000000011001> |
| Central inflammatory - Encephalitis | Encephalitis, with positive CSF PCR for SARS CoV-2 | 1 | <https://doi.org/10.1016/j.jstrokecerebrovasdis.2021.105915> |
| Central inflammatory - Vasculitis | Intracranial vasculitis | 1 | <https://dx.doi.org/10.1136/jnnp-2020-324291> |
| Peripheral nerve - Inflammatory | Guillain-Barre syndrome | 25 | <https://doi.org/10.1101/2020.07.24.20161471> |

**Supplementary Table 1.**Cases included in this study which have been published elsewhere.

| **Symptom** | **n (%)** |
| --- | --- |
| Cough | 139 (67) |
| Fever | 172 (73) |
| Rhinorrhoea | 24 (13) |
| Sore throat | 18 (11) |
| Headache | 37 (22) |
| Anosmia | 15 (13) |
| Loss of taste | 12 (10) |
| Chest pain | 20 (11) |
| Wheeze | 26 (14) |
| Shortness of breath | 134 (61) |
| Lethargy | 124 (68) |
| Arthralgia | 13 (10) |
| Myalgia | 42 (27) |
| Diarrhoea | 34 (18) |
| Abdominal pain | 24 (13) |
| Vomiting | 23 (12) |
| Other | 37 (21) |

**Supplementary Table 2.**Reported non-neurological symptoms in all patients.

| **Cerebrovascular cases (n=131)** | | | **<60 years (n=35)** | **≥ 60 years** **(n=96)** | **p value** |
| --- | --- | --- | --- | --- | --- |
| Female sex, N (%) |  | | 12 (34) | 39 (41) | ns^b^ |
| Onset relative to respiratory symptoms, median (IQR)  (n=130) | | | 10 (0-18) | 0 (-7 – 7) | <0·001^a^ |
| At least one co-morbidity increasing stroke risk, N (%) | |  | 16 (67) | 77 (88) | 0·016^b^ |
| ICU admission, N (%) |  | | 14 (41) | 15 (16) | 0·002^b^ |
| Non-CNS thrombotic event, N (%) |  | | 6 (18) | 8 (8) | ns^b^ |
| Admission bloods, median (IQR) | CRP (n=124) | | 73 (11-198) | 41 (6-114) | ns^a^ |
|  | Platelets (n=129) | | 245 (175-322) | 231 (174-304) | ns^a^ |
|  | Lymphocytes (n=129) | | 1 (0·8-1·7) | 1 (0·6-1·5) | ns^a^ |
| Subtype, N (%) | Ischaemic | | 29 (83) | 76 (79) | ns^b^ |
|  | Ischaemic: LVO | | 15 (52) | 39 (51) | ns^b^ |
|  | Ischaemic: MVO | | 9 (31) | 11 (14) | 0·053^b^ |
|  | Haemorrhagic | | 2 (6) | 19 (20) | 0·052^b^ |
|  | CVST | | 4 (11) | 1 (1) | 0·006^b^ |
| mRS score, median (IQR) | Nadir (n=129) | | 4 (3-5) | 5 (4-5) | ns^a^ |
|  | Outcome (n=128) | | 3 (1-5) | 4 (3-6) | ns^a^ |

**Supplementary Table 3.**Differences in clinical characteristics between young (age <60 years) and old (>60 years) strokes. Co-morbidities increasing stroke risk were defined as hypertension, atrial fibrillation, diabetes mellitus, congestive heart failure and previous cerebrovascular disease. ‘LVO’ refers to large vessel occlusion, ‘MVO’ refers to multi-vessel occlusion. P-values derived from Mann-Whitney^a^and Chi-squared^b^ tests

|  | | | **Cerebrovascular event** | **Central inflammatory** | **Delirium** | **Psychiatric** | **Other CNS** | **Peripheral** **neuropathy** |
| --- | --- | --- | --- | --- | --- | --- | --- | --- |
| Age in years, n (%) | 20-29 | | 1 (0·8) | 0 (0) | 1 (4) | 2 (8) | 0 (0) | 2 (5) |
|  | 30-39 | | 2 (1·5) | 4 (16) | 4 (14) | 1 (4) | 3 (17·6) | 1 (2) |
|  | 40-49 | | 17 (13·0) | 4 (16) | 0 (0) | 3 (12) | 3 (17·6) | 8 (20) |
|  | 50-59 | | 15 (11·5) | 8 (32) | 6 (22) | 8 (32) | 5 (29·4) | 15 (37) |
|  | 60-69 | | 24 (18·3) | 7 (28) | 4 (14) | 2 (8) | 3 (17·6) | 11 (27) |
|  | 70-79 | | 29 (22·1) | 1 (4) | 9 (32) | 7 (28) | 1 (5·9) | 3 (7) |
|  | 80-89 | | 28 (21·4) | 1 (4) | 2 (7) | 2 (8) | 2 (11·8) | 1 (2) |
|  | >90 | | 15 (11·5) | 0 (0) | 2 (7) | 0 (0) | 0 (0) | 0 (0) |
| Sex, n (%) | Male | | 80 (61) | 22 (88) | 19 (68) | 11 (44) | 9 (53) | 31 (76) |
|  | Female | | 51 (39) | 3 (12) | 9 (32) | 14 (56) | 8 (47) | 10 (24) |
| Ethnicity, n (%) | Asian | | 10 (8) | 3 (12) | 2 (7) | 3 (12) | 1 (6) | 4 (10) |
|  | Black | | 6 (4) | 3 (12) | 4 (14) | 3 (12) | 2 (12) | 3 (7) |
|  | White | | 107 (82) | 17 (68) | 17 (61) | 15 (60) | 12 (70) | 28 (68) |
|  | Mixed | | 1 (1) | 1 (4) | 0 (0) | 1 (4) | 0 (0) | 0 (0) |
|  | Unknown | | 7 (5) | 1 (4) | 5 (18) | 3 (12) | 2 (12) | 6 (15) |
| COVID diagnosis, n (%) | Confirmed or probable | | 130 (99) | 22 (88) | 27 (93) | 20 (80) | 16 (94) | 25 (61) |
|  | Possible | | 1 (1) | 3 (12) | 2 (7) | 5 (20) | 1 (6) | 16 (39) |
| ICU admission, n (%) | Yes | | 29 (22) | 13 (52) | 8 (29) | 4 (16) | 10 (59) | 12 (29) |
|  | No | | 100 (76) | 11 (44) | 19 (68) | 21 (84) | 7 (41) | 13 (32) |
|  | Unknown | | 2 (2) | 1 (4) | 1 (3) | 0 (0) | 0 (0) | 16 (39) |
| Ventilation required, n (%) | None | | 100 (76) | 10 (40) | 18 (64) | 18 (72) | 6 (35) | 13 (32) |
|  | NIV | | 6 (5) | 1 (4) | 2 (7) | 5 (20) | 0 (0) | 1 (2) |
|  | Invasive | | 24 (18) | 13 (52) | 7 (25) | 2 (8) | 10 (59) | 11 (27) |
|  | Unknown | | 1 (1) | 1 (4) | 1 (4) | 0 (0) | 1 (6) | 16 (39) |
| Pre-COVID-19 frailty score, median (IQR) | | | 3 (2-6) | 2 (1-2) | 3 (2-5) | 3 (2-4) | 2 (2-4) | 2 (1-2) |
| At least one co-morbidity, n (%) | |  | 115 (88) | 18 (78) | 21 (75) | 16 (64) | 14 (82) | 12 (63) |
| Number of co-morbidities, median (IQR) | |  | 3 (1-4) | 2 (1-4) | 3 (0-5) | 1 (0-3) | 2 (1-4) | 1 (0-2) |
| Admission GCS, median IQR) | |  | 15 (14-15) | 15 (14-15) | 15 (14-15) | 15 (14-15) | 14 (14-15) | 15 (15-15) |
| Fever, n (%) | |  | 71 (63) | 22 (92) | 23 (85) | 16 (80) | 10 (77) | 30 (77) |
| Admission WCC, median (IQR) | |  | 8·0 (6·0-12·0) | 8·0 (6·0-12·0) | 9·0 (6·0-10·0) | 7·0 (6·0-11·0) | 9·5 (5-13) | 8·0 (6·0-11·0) |
| Admission CRP, median (IQR) | |  | 42 (7-145) | 61 (7-199) | 46 (15-158) | 42 (11-86) | 64 (12-140) | 13 (10-28) |
| Any non-neurological, non-respiratory complication, n (%) | |  | 46 (36) | 15 (68) | 13 (46) | 9 (36) | 9 (53) | 9 (41) |
| mRS at nadir, median (IQR) | |  | 5 (3-5) | 5 (3-5) | 5 (3-5) | 3 (2-4) | 5 (4-5) | 3 (2-4) |
| mRS at outcome, median (IQR) | |  | 4 (3-6) | 3 (2-4) | 3 (1-5) | 2 (1-3) | 3 (1-6) | 2 (1-4) |
| Improvement in mRS score, n (%) | |  | 50 (39) | 17 (77) | 15 (58) | 18 (72) | 11 (69) | 14 (74) |
| Admission length in days, median (IQR) | |  | 21 (8-43) | 45 (22-73) | 26 (14-54) | 6 (3-26) | 32 (16-53) | 23 (2-59) |
| Death n (%) | |  | 46 (36) | 0 (0) | 6 (23) | 1 (4) | 4 (25) | 0 (0) |

**Supplementary Table 4.**Patient demographics and clinical parameters by primary diagnostic categories. mRS refers to modified Rankin Scale. Pre-COVID-19 frailty score refers to Rockwood frailty score.

|  | **With delirium** | **Without delirium** | **p** |
| --- | --- | --- | --- |
| N | 38 | 229 |  |
| Age by decade, median (IQR) | 60-69 (50-79) | 60-69 (50-79) | 0·92^a^ |
| Frailty score, median (IQR) | 3 (2-5) | 3 (2-5) | 0·35^a^ |
| Number of comorbidities, median (IQR) | 3 (1-5) | 2 (1-4) | 0·41^a^ |
| Preceding psychiatric illness, n (%) | 6 (16) | 16 (8) | 0·15^#^ |
| Anticholinergic drugs, n (%) | 5 (17) | 14 (10) | 0·25^#^ |
| Fever, n (%) | 28 (85) | 144 (71) | 0·095^#^ |
| Admission WCC, median (IQR) | 9·0 (6·5-12·0) | 8·0 (6·0-12·0) | 0·50^a^ |
| Admission CRP, median (IQR) | 51 (15-137) | 40 (7-140) | 0·36^a^ |
| Need for intensive care, n (%) | 13 (35) | 63 (30) | 0·53^#^ |

**Supplementary Table 5.** Risk factors for delirium. Analysis was performed in all patients with delirium (not just cases where delirium was a primary diagnosis), versus the rest of the patients. Drugs were assessed for their capacity to cause delirium using the anticholinergic effect on cognition scale (Bishara 2016 - <https://doi.org/10.1002/gps.4507>); in cases where the total number of points on the anticholinergic effect on cognition scale for all listed drugs exceeded 2, a clinically significant anticholinergic effect was assumed. ^a^Mann-Whitney U test ^#^χ^2^ test

| **Age (years)** | **Sex** | **Ethnicity** | **Diagnoses** | **Clinical frailty scale** | **Neurological comorbidity** | **Medical comorbidities** | **Cardiac complications** | **Renal complications** | **ICU** | **Medication** | **EEG results** | **mRS at nadir** |
| --- | --- | --- | --- | --- | --- | --- | --- | --- | --- | --- | --- | --- |
| 31 to 40 | F | Unknown | Provoked seizure | Well |  | Hypothyroid, migraine, depression |  |  | N | Levothyroxine | Normal | 1 |
| 31 to 40 | M | White | Status epilepticus, multiple seizures | Well |  | T2DM |  |  | Y | Levetiracetam, metformin | Normal | 4 |
| 31 to 40 | M | White | Unexplained coma with tetraparesis, pupillary and eye movement abnormalities | Very Fit |  | Hypertension |  |  | Y | Aspirin, candesartan, esomeprazole | Not performed | 5 |
| 41 to 50 | M | Black or African or Caribbean or Black British | Encephalopathy with multiple seizures | Very Fit | Cerebrovascular disease | Hypertension |  |  | N |  | No results provided | 4 |
| 41 to 50 | M | Asian or Asian British | Encephalopathy with myoclonus | Well |  |  | Recurrent atrial flutter requiring DC cardioversion | Acute kidney injury requiring haemodialysis | Y | Rivaroxaban | Slow background suggesting mild encephalopathy | 5 |
| 51 to 60 | M | White | Encephalopathy with choreoathetosis and myoclonus | Very Fit |  |  | Right heart failure |  | Y | Olanzapine | Not performed | 5 |
| 51 to 60 | F | Black or African or Caribbean or Black British | Encephalopathy with myoclonus | Vulnerable |  | Polycystic kidney disease, recent renal transplant, Toxoplasmosis, CMV viraemia, T2DM | Cardiac arrest - asystole |  | Y | Multiple courses of antibiotics, heparin, warfarin, oxycodone, paracetamol, tacrolimus, prednisolone, mycophenolate, omeprazole, amlodipine, bisoprolol, lamivudine, pyridoxine, valganciclovir, aspirin | Mild slowing. No cortical correlates to twitches | 5 |
| 51 to 60 | F | Unknown | Severe encephalopathy | Very Fit |  | Mild asthma | Prolonged QTC | Acute kidney injury | Y | Multiple courses of IV antibiotics and antifungals, gabapentin, dexamethasone | Not performed | 6 |
| 51 to 60 | M | White | Encephalopathy with coma and seizure | Vulnerable | LD, ASD, schizophrenia |  |  |  | Y | Clozapine, valproic acid, cholecalciferol, procyclidine, enoxaparin | Slow anterior emphasis, with bi-hemispheric dysfunction | 5 |
| 61 to 70 | M | White | Encephalopathy with multiple seizures | Managing Well |  | Hypertension, pulmonary embolism, pancreatitis, T2DM |  |  | Y | Insulin, atorvastatin, carbocisteine, creon, lansoprazole, thyroxine, metformin, ramipril, Rivoroxaban | Mild encephalopathy | 5 |
| 61 to 70 | F | White | Encephalopathy with multiple seizures | Severely Frail | Frontotemporal dementia; previous subdural haematoma | NIDDM |  |  | N | Metformin, atorvastatin, citalopram | Not performed | 5 |
| 61 to 70 | M | White | Encephalopathy with seizure and left sided weakness | Mildly Frail | Dementia | COPD, alcohol abuse |  |  | N | Omeprazole, propranolol, steroid inhaler | Not performed | 6 |
| 81 to 90 | F | White | Encephalopathy with non-convulsive status epilepticus | Severely Frail | Stroke, epilepsy, dementia | Hypercholesterolaemia |  |  | N | Levetiracetam, atorvastatin, sertraline, clopidogrel | Moderate encephalopathy with PLEDs. Status without electrographic recovery. | 6 |

**Supplementary Table 6.**Demographic and clinical characteristics of 13 cases with severe encephalopathy, outside the clinical definition of delirium.

**Abbreviations**: DC = direct current, QTc = corrected QT interval, LD = learning disability, ASD = autism spectrum disorder T2DM = type 2 diabetes mellitus, COPD = chronic obstructive pulmonary disorder, CMV = cytomegalovirus, NIDDM = non-insulin-dependent diabetes mellitus, IV = intravenous.

| **Primary diagnostic category** | **Improvement in mRS n (%)** | **z test** |
| --- | --- | --- |
| Cerebrovascular event | 50 (39) | p<0.001 |
| Central inflammatory | 17 (77) | p<0.02 |
| Delirium | 15 (58) | NS |
| Psychiatric | 18 (72) | p<0.05 |
| Other CNS | 11 (69) | NS |
| Peripheral neuropathy | 14 (74) | NS |

**Supplementary Table 7. Clinical outcome:** improvement in neurological function from nadir (within patient improvement between nadir and follow-up mRS – yes / no), as measured with the dichotomised mRS, differed across primary diagnostic groups (χ^2^ (5, N = 236) = 23.9, p < 0.001). z tests for independent proportions were two-tailed.

| **Variable** | **Missing data %** |
| --- | --- |
| Age (10-year age groups) | 0 |
| Sex at birth (Male) | 0 |
| Non-white ethnic group | 9 |
| Clinical frailty scale (Rockwood) | 12.7 |
| Pre-existing neurological disease | 12.7 |
| Hypertension | 3 |
| Diabetes | 2.2 |
| Log_10_ white cell count at admission | 16.5 |

**Supplementary Table 8. Clinical outcome:** percentage of data missingness in the variables used to model clinical outcome

**SUPPLEMENTARY FIGURES**


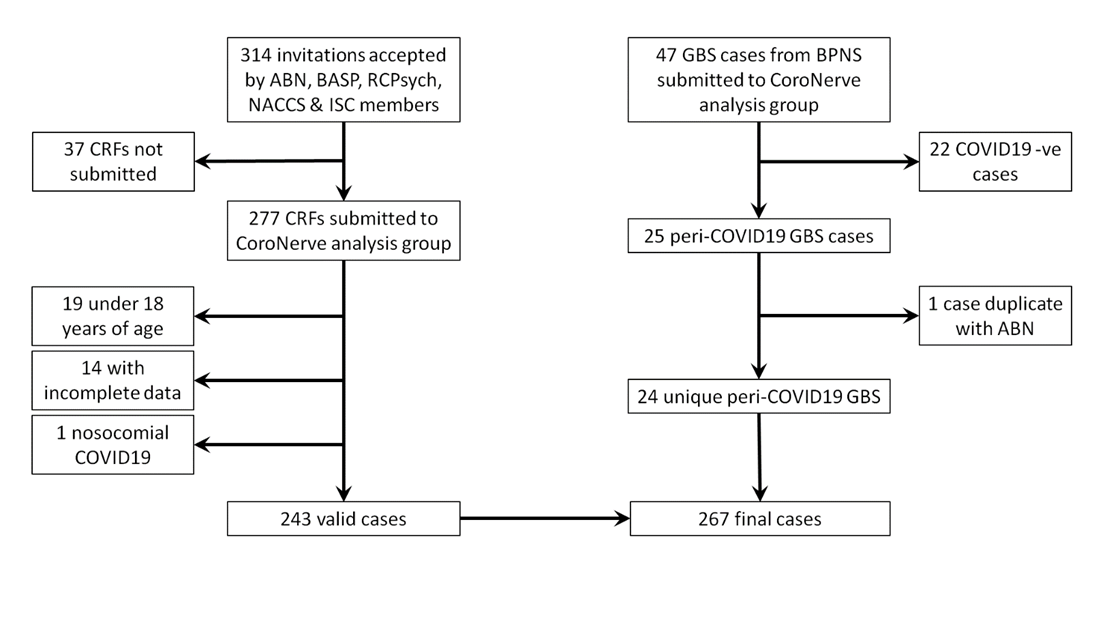


**Supplementary Figure 1.** Recruitment flow diagram

**
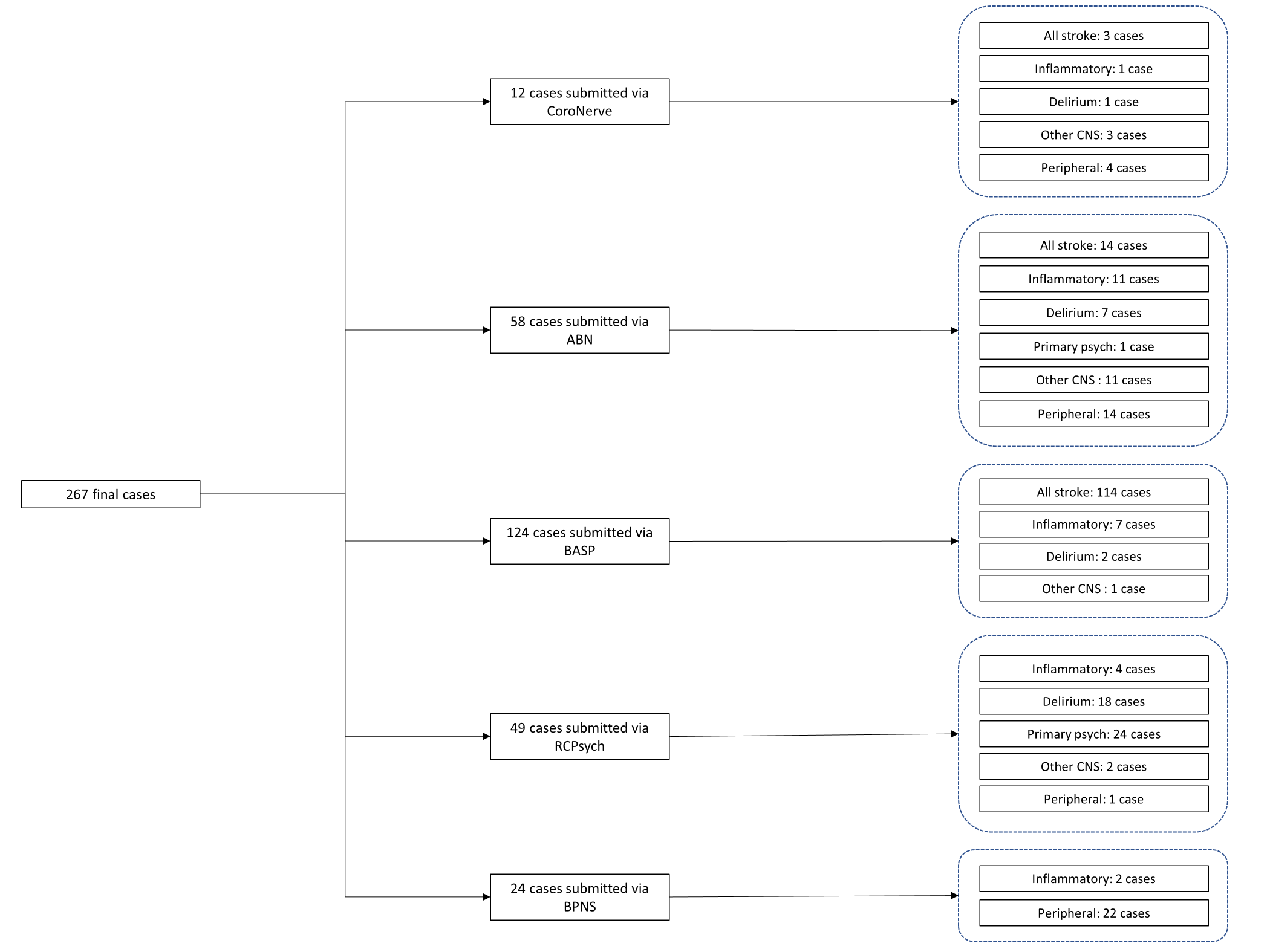
**

**Supplementary Figure 2.** Provenance of final cases by professional association platform. Association of British Neurologists; BASP: British Association of Stroke Physicians; RCPsych: Royal College of Psychiatrists; BPNS: British Peripheral Nerve Society

**
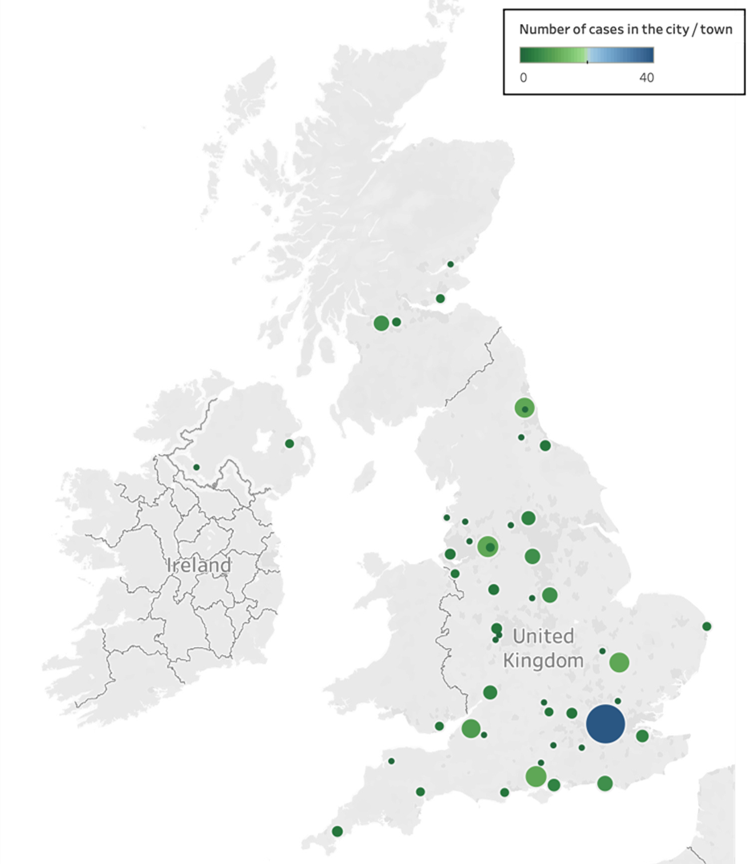
**

**Supplementary Figure 3.** Geographical spread of cases in the study, for those whose hospital details were available (n=219).
