# Supplementary material for: The wide spectrum of Covid-19 neuropsychiatric complications within a multidisciplinary center
Source: Brain Commun. 2021 Jun 17:fcab135. doi: 10.1093/braincomms/fcab135 (PMC8344449; doi:10.1093/braincomms/fcab135)

Supplementary Figure 1

Decision tree trained to predict whether a patient who went to ICU had an ICU neuropathy or not. The depth of the tree was imposed to 4 and trained through entropy minimization. 114 patients went to ICU and 60 had an ICU neuropathy.

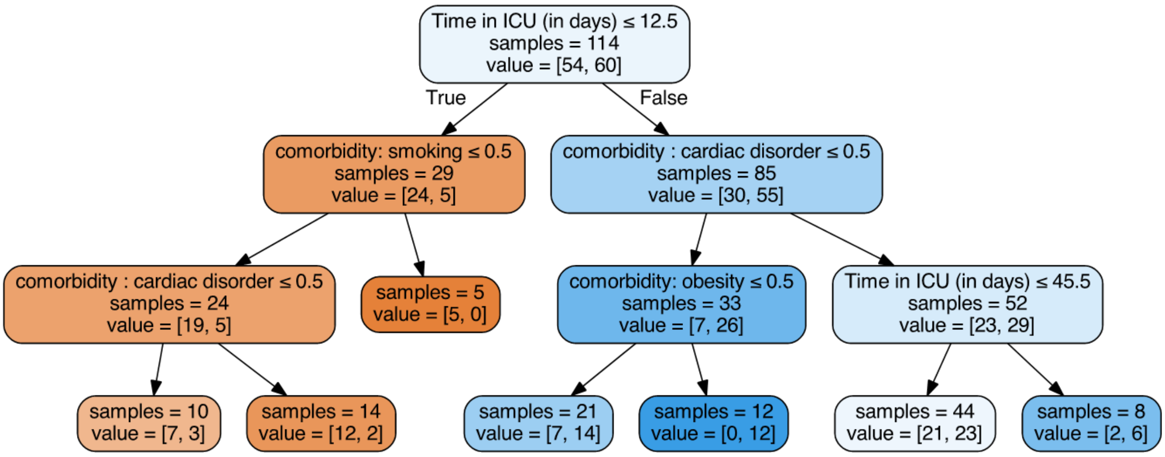

Supplement: Supplementary file 1 [file fcab135_supplementary_data.zip › suppl_data/Supplementary Figure .pdf]
