# Supplementary material for: The wide spectrum of Covid-19 neuropsychiatric complications within a multidisciplinary center
Source: Brain Commun. 2021 Jun 17:fcab135. doi: 10.1093/braincomms/fcab135 (PMC8344449; doi:10.1093/braincomms/fcab135)
Supplement: Supplementary file 1 [file fcab135_supplementary_data.zip › suppl_data/The Coco-Neurosciences Study Group.pdf]

The Coco-Neurosciences Study Group:

Steering Committee (Pitié-Salpêtrière Hospital, Paris): Cecile Delorme, Jean-Christophe Corvol, Jean-Yves Delattre, Stephanie Carvalho, Sandrine Sagnes. **Scientific Committee** (Pitié-Salpêtrière Hospital, Paris): Bruno Dubois, Vincent Navarro, Celine Louapre, Tanya Stojkovic, Ahmed Idbaih, Charlotte Rosso, David Grabli, Ana Zenovia Gales, Bruno Millet, Benjamin Rohaut, Eleonore Bayen, Sophie Dupont, Gaelle Bruneteau, Stephane Lehericy, Danielle Seilhean, Alexandra Durr, Foudil Lamari, Marion Houot, Vanessa Batista Brochard. **Principal investigators**: Pitié-Salpêtrière Hospital (Paris): Sophie Dupont, Catherine Lubetzki, Danielle Seilhean, Pascale Pradat-Diehl, Charlotte Rosso, Khe Hoang-Xuan, Bertrand Fontaine, Lionel Naccache, Philippe Fossati, Isabelle Arnulf, Alexandra Durr, Alexandre Carpentier, Stephane Lehericy, Yves Edel; Rothschild Hospital (Paris): Gilberte Robain, Philippe Thoumie; Avicenne Hospital (Bobigny): Bertrand Degos; Sainte-Anne Hospital (Paris): Tarek Sharshar; Saint-Antoine Hospital (Paris): Sonia Alamowitch, Emmanuelle Apartis-Bourdieu, Charles-Siegried Peretti; Saint-Louis Hospital (Paris): Renata Ursu; Tenon Hospital (Paris): Nathalie Dzierzynski; Charles Foix Hospital (Ivry): Kiyoka Kinugawa Bourron, Joel Belmin, Bruno Oquendo, Eric Pautas, Marc Verny. **Co-investigators**: Pitié-Salpêtrière Hospital (Paris): Cecile Delorme, Jean-Christophe Corvol, Jean-Yves Delattre, Yves Samson, Sara Leder, Anne Leger, Sandrine Deltour, Flore Baronnet, Ana Zenovia Gales,Stephanie Bombois, Mehdi Touat, Ahmed Idbaih, Marc Sanson, Caroline Dehais, Caroline Houillier, Florence Laigle-Donadey, Dimitri Psimaras, Agusti Alenton, Nadia Younan, Nicolas Villain, David Grabli, Maria del Mar Amador, Gaelle Bruneteau, Celine Louapre, Louise-Laure Mariani, Nicolas Mezouar, Graziella Mangone, Aurelie Meneret, Andreas Hartmann, Clement Tarrano, David Bendetowicz, Pierre-François Pradat, Michel Baulac, Sara Sambin, Phintip Pichit, Florence Chochon, Adele Hesters, Bastien HerlinAn Hung Nguyen, Valerie Procher, Alexandre Demoule, Elise Morawiec, Julien Mayaux, Morgan Faure, Claire Ewenczyk, Giulia Coarelli, Anna Heinzmann, Tanya Stojkovic, Marion Masingue, Guillaume Bassez, Vincent Navarro, Isabelle An, Yulia Worbe, Virginie Lambrecq, Rabab Debs, Esteban Munoz Musat, Timothee Lenglet, Virginie Lambrecq, Aurelie Hanin, Lydia Chougar, Nathalia Shor, Nadya Pyatigorskaya, Damien Galanaud, Delphine Leclercq, Sophie Demeret, Benjamin Rohaut, Albert Cao, Clemence Marois, Nicolas Weiss, Salimata Gassama, Loic Le Guennec, Vincent Degos, Alice Jacquens, Thomas Similowski, Capucine Morelot-Panzini, Jean-Yves Rotge, Bertrand Saudreau, Bruno Millet, Victor Pitron, Nassim Sarni, Nathalie Girault, Redwan Maatoug, Ana Zenovia Gales, Smaranda Leu, Eleonore

Bayen, Lionel Thivard, Karima Mokhtari, Isabelle Plu; Sainte-Anne Hospital (Paris): Bruno Gonçalves; Saint-Antoine Hospital (Paris): Laure Bottin, Marion Yger; Rothschild Hospital (Paris): Gaelle Ouvrard, Rebecca Haddad; Charles Foix Hospital (Ivry): Flora Ketz, Carmelo Lafuente, Christel Oasi. **Other Contributors**: **Associated centers** (Lariboisière Hospital, Paris): Bruno Megabarne, Dominique Herve; **Clinical Research Associates** (ICM, Pitié-Salpêtrière Hospital, Paris): Haysam Salman, Armelle Rametti-Lacroux, Alize Chalançon, Anais Herve, Hugo Royer, Florence Beauzor, Valentine Maheo, Christelle Laganot, Camille Minelli, Aurelie Fekete, Abel Grine, Marie Biet, Rania Hilab, Aurore Besnard, Meriem Bouguerra, Gwen Goudard, Saida Houairi, Saba Al-Youssef, Christine Pires, Anissa Oukhedouma, Katarzyna Siuda-Krzywicka, Tal Seidel Malkinson; (Saint-Louis Hospital, Paris): Hanane Agguini; **Data Manager** (ICM, Paris): Safia Said; **Statistician** (ICM, Paris): Marion Houot.
